# Nutritional Status According to the Short-Form Mini Nutritional Assessment (MNA-SF) and Clinical Characteristics as Predictors of Length of Stay, Mortality, and Readmissions Among Older Inpatients in China: A National Study

**DOI:** 10.3389/fnut.2022.815578

**Published:** 2022-01-25

**Authors:** Hongpeng Liu, Jing Jiao, Minglei Zhu, Xianxiu Wen, Jingfen Jin, Hui Wang, Dongmei Lv, Shengxiu Zhao, Xiang Sun, Xinjuan Wu, Tao Xu

**Affiliations:** ^1^Department of Nursing, Chinese Academy of Medical Sciences—Peking Union Medical College, Peking Union Medical College Hospital (Dongdan Campus), Beijing, China; ^2^Department of Geriatrics, Chinese Academy of Medical Sciences—Peking Union Medical College, Peking Union Medical College Hospital (Dongdan Campus), Beijing, China; ^3^Department of Nursing, Sichuan Provincial People's Hospital, Chengdu, China; ^4^Department of Nursing, The Second Affiliated Hospital Zhejiang University School of Medicine, Hangzhou, China; ^5^Department of Nursing, Tongji Medical College, Tongji Hospital, Huazhong University of Science and Technology, Wuhan, China; ^6^Department of Nursing, The Second Affiliated Hospital of Haerbin Medical University, Haerbin, China; ^7^Department of Nursing, Qinghai Provincial People's Hospital, Xining, China; ^8^Department of Epidemiology and Statistics, Institute of Basic Medical Sciences, Peking Union Medical College, Chinese Academy of Medical Sciences and School of Basic Medicine, Beijing, China

**Keywords:** malnutrition parameters, mortality, length of stay, readmission, older inpatients, nutritional epidemiology

## Abstract

**Background:**

Studies are scarce in China that explore the association of nutritional status, measured using the Short-Form Mini Nutritional Assessment (MNA-SF) and biochemical data, on adverse clinical outcomes among older inpatients. In this study, we aimed to determine the prevalence of malnutrition in tertiary hospitals of China and the associations between malnutrition and adverse clinical outcomes.

**Methods:**

This prospective study involved 5,516 older inpatients (mean age 72.47 ± 5.77 years) hospitalized in tertiary hospitals between October 2018 and February 2019. The tertiary hospitals refer to the hospital with more than 500 beds and can provide complex medical care services. The MNA-SF was used to assess nutritional status. Multiple logistic regression and negative binomial regression were used to analyze the relationship between nutritional parameters and risk of hospital length of stay (LoS), mortality, and rehospitalization.

**Results:**

We found that 46.19% of hospitalized patients had malnutrition or malnutrition risk, according to the MNA-SF. Death occurred in 3.45% of patients. MNA-SF scores 0–7 (odds ratio [OR] 5.738, 95% confidence interval [CI] 3.473 to 9.48) were associated with a six-fold higher likelihood of death, and scores 8–11 (OR 3.283, 95% CI 2.126–5.069) with a three-fold higher likelihood of death, compared with MNA-SF scores 12–14 in the logistic regression model, after adjusting for potential confounders. A low MNA-SF score of 0–7 (regression coefficient 0.2807, 95% CI 0.0294–0.5320; *P* < 0.05) and a score of 8–11 (0.2574, 95% CI 0.0863–0.4285; *P* < 0.01) was associated with a significantly higher (28.07 and 25.74%, respectively) likelihood of increased LoS, compared with MNA-SF score 12–14. MNA-SF scores 0–7 (OR 1.393, 95% CI 1.052–1.843) and 8–11 (OR 1.356, 95% CI 1.124–1.636) were associated with a nearly 1.5-fold higher likelihood of 90-day readmission compared with MNA-SF scores 12–14 in the logistic regression model. Moreover, hemoglobin level, female sex, education level, former smoking, BMI 24–27.9 kg/m^2^, age 75 years and above, and current alcohol consumption were the main factors influencing clinical outcomes in this population.

**Conclusions:**

Malnutrition increases the risk of hospital LoS, mortality, and 90-day readmission. The use of nutritional assessment tools in all hospitalized patients in China is needed. The MNA-SF combined with hemoglobin level may be used to identify older inpatients with a high risk of adverse clinical outcomes. These findings may have important implications for the planning of hospital services.

## Introduction

With the rapid development of health science and the global economy, the life span of the world's population is increasing (1–3). By 2050, it is estimated that 16% of the world's population would be aged 65 years and older (4), with 80% of those living in low- and middle-income countries (5). China, as the world's second-largest economy, has the world's largest population of 1.44 billion (19% of the world's population) and is rapidly becoming an aging nation (6–8).

Older adults are likely to have poor nutritional status and decreased quality of life (8–10). Nutritional deficits in the elderly can be caused by insufficient nutrient and energy intake (11). A few age-related pathophysiological, psychosocial, and pharmacological factors determine changes in dietary habits, as well as intake and use of nutrients, leading to specific deficits (12). The adverse effects of malnutrition or malnutrition risk on health substantially affect the quality of life by increasing the risk of physical frailty, disability, mortality, and deterioration during hospitalization (15, 19).

The prevalence of hospital malnutrition or the risk of malnutrition in elderly patients is considerable (30–50%) (11, 13, 14). This is primarily due to challenges in identifying and appropriately managing at-risk patients (15). As the European Society for Parenteral and Enteral Nutrition (ESPEN) recommends (16), Nutrition Risk Screening (NRS 2002) should be used to screen undernutrition in all inpatients. Several nutritional measures have been developed to determine nutritional status among inpatients in China, including the NRS 2002 and Short-Form Mini Nutritional Assessment (MNA-SF) (14, 17). However, appropriate nutritional risk screening is not performed in many Chinese hospitals, only in some large-scale nationwide, provincial, and municipal tertiary hospitals with more than 500 beds (18), where nutritional risk screening is mandatory.

Previous reports have suggested that malnutrition is associated with increased mortality among older residents of nursing homes (19). Other studies have used various nutritional screening tools, including the NRS 2002 and Malnutrition Universal Screening Tool, to estimate nutritional status and adverse clinical outcomes in the general hospitalized population (17, 20, 21) and in surgical patients (10, 22, 23), or these have used hospital data only or a smaller sample size (10, 24–27).

The European Society for Parenteral and Enteral Nutrition (ESPEN) suggests the use of NRS 2002 and Malnutrition Universal Screening Tool, whereas for older populations ESPEN recommends the use of the Mini Nutritional Assessment (MNA) either in its full or short form (MNA-SF) (28). Previous studies also indicated that the MNA-SF is a valid instrument with good specificity and sensitivity for the diagnosis of malnutrition, and this tool is specific for the older population (28–30). Nevertheless, few studies have reported on the prevalence of malnutrition in Asia hospitals (31), and studies are scarce in China that explore the associations between nutritional status, measured using the MNA-SF and biochemical data, on adverse clinical outcomes among older inpatients. To address this issue, we used data from a cohort study to examine the implementation of nutritional risk screening. We also assessed the prevalence, determinants, and associations between nutritional status and hospital length of stay (LoS), mortality, and readmission.

## Methods

### Participants and Data Collection

Participants were inpatients aged 65 years and older from an ongoing, prospective, large-scale cohort study of elder patients hospitalized in tertiary hospitals in China (Eastern: Zhejiang Province; South-Central: Hubei Province; southwest: Sichuan Province; Northeast: Heilongjiang Province; Northern: Beijing municipality/city; Northwest: Qinghai Province). Details can be found elsewhere (14, 32). Eligible study subjects are recruited from neurology, surgical, orthopedics departments, intensive care unit (ICU), as well as internal medicine of selected hospitals, are consecutively enrolled. The surveys are managed by trained nurses using a structured Case Report Form (CRF). Our research team developed manuals for the project survey and operation process. To ensure data quality, the nurses received training and test before they apply the assessment to the patients. All CRF results were reviewed by the head nurse. The research team also developed a quality control team, and a communication platform based on the WeChat App to guarantee timely feedback. If the participant is unable to answer questions on his or her own, proxy interviewees (usually spouses or other legal guardians) are interviewed.

The current study was based on baseline survey data collected from October 2018 to February 2019. During this period, there were 9,996 hospitalizations; of these, 8,326 patients had complete data at 90-day follow-up, and 5,516 had an LoS of at least 2 days or had initial biochemical data during hospitalization. Thus, the study subjects included a total of 5,516 patients aged 65 years and older.

### Measurements

Nutritional status was assessed using the MNA-SF, a six-item instrument with scores ranging from 0 to 14 points (19). The MNA-SF has been validated and shows good specificity and sensitivity for the diagnosis of malnutrition, mainly in older adults (10, 29). For the purpose of our study, participants were categorized into a group with normal nutritional status (12–14 points), the patient at risk of malnutrition (8–11 points), or malnourished patients (0–7 points) (33). The MNA-SF has been verified in the Chinese elderly and has extraordinary test characteristics (14, 34).

Participants' height (cm) was measured to the nearest 1 mm using a stadiometer, while weight (kg) was measured to the nearest 0.1 kg using a digital electronic chair scale. Patients were weighed while wearing light clothing and without shoes. Considering most of the ICU patients were immobility, the body weight and height were recorded when they were first admitted to the general ward, and this data can be synchronized to the ICU nursing information system. Therefore, our research team record their body weight and height according to the information system. Body mass index (BMI) was calculated as body weight divided by height (in meters) squared (kg/m^2^) (35) and was used to classify patients of underweight (<18.5 kg/m^2^), normal (18.5–23.9 kg/m^2^), overweight (24–27.9) kg/m^2^), and obese greater or equal to 28 kg/m^2^) (36, 37).

The following parameters were also analyzed: age, alcohol consumption, smoking (former smokers refer to at least 6 months without smoking), sex, education level, ethnicity, and marital status. All biochemical parameters included in the analysis (such as hemoglobin, serum albumin, blood urea nitrogen, and creatinine) were the first determinations during patients' respective hospitalizations (21).

### Measured Outcomes

The following outcomes were measured: death (record all-cause mortality within 90 days, which consists of in-hospital deaths), LoS (duration of hospitalization) (38), and nonelective readmission (second and subsequent hospitalizations during the period analyzed) during the 90 days following discharge.

### Bioethics

This study was ethically approved by the review board of Peking Union Medical College Hospital (S-K540). All patients participating in this study provided written informed consent. If patients had cognitive decline, the investigator interviewed their proxy respondents and obtained their consent for the patient to participate in this study. Patients were excluded if they were persistently unconscious, or if their proxy respondents were unable to provide effective information.

### Statistical Analyses

Statistical analysis using SAS 9.4 software (SAS Institute Inc., Cary, NC, USA). Continuous variables are summarized as mean and standard deviation (SD). Categorical variables were summarized as counts and percentages. Bivariate analyses were performed using the χ^2^ test or Fisher's exact test for qualitative variables and the Student *t-*test, analysis of variance, or Kruskal–Wallis test for quantitative variables. We ran separate models for each parameter of nutritional status in all analyses, owing to the collinearity between them. Both logistic and negative binomial regression analyses were conducted. The strength of the associations was estimated as odds ratios (ORs) for the logistic models, and as the regression coefficient for negative binomial regression, with 95% confidence intervals (95% CIs). A *P*-value of <0.05 was considered statistically significant.

## Results

### Participant Characteristics

In Table 1, the mean age of the included patients was 72.47 ± 5.77 years at baseline, and 59.77% of participants (3,297/5,516) were men. Approximately 54% of patients had a middle school education and above, and 90.1% were married. A major proportion of respondents were of Han nationality (94.65%). More than half of the participants were non-smokers (65.07%) and non-drinkers (74.85%). The average BMI was 23.56 ± 3.52 kg/m^2^, and approximately half (49.02%) had a BMI between 18.5 and 23.9 kg/m^2^. 40.08% of patients were from medicine departments and only 5.73% of patients were from ICU. According to the MNA-SF, 46.19% (2,548/5,516) of hospitalized individuals were at risk of malnutrition or malnourishment (Table 2). Patients who were malnourished were with higher mortality and 90-day readmission, and longer LoS. 11.89% (656/5,516) of malnourished patients had low serum albumin [cut-off 35 g/L for serum albumin levels (19)] compared with the well-nourished patients or patients at risk of malnutrition. The remaining data for hospitalizations analyzed according to MNA-SF values for malnutrition, malnutrition risk, or normal, are presented in Tables 1, 2.

**Table 1:** Demographic and clinical characteristics of patients in relation to MNA-SF score on admission.

**Parameter**	**Cases (*N* = 5,516)**
Age (years)	
Age ranges	72.47 ± 5.77
<75	3,799 (68.87)
≥75	1,717 (31.13)
Sex (*n*, %)	
Male gender	3,297 (59.77)
Female gender	2,219 (40.23)
Education (*n*, %) (*n* = 5,514)	
University	754 (13.67)
Middle school	2,203 (39.95)
Primary school	1,644 (29.82)
Illiterate	913 (16.56)
Marital status (*n*, %) (*n* = 5,510)	
Married	4,964 (90.1)
Divorced or widowed	546 (9.91)
Ethnicity (*n*, %)	
Han	5221 (94.65)
Others	295 (5.35)
Smoking (*n*, %)	
Current smoker	641 (11.62)
Former smoker	1,286 (23.31)
Non-smoker	3,589 (65.07)
Alcohol consumption (*n*, %)	
Current drinker	686 (12.44)
Former drinker	701 (12.71)
Non-drinker	4,129 (74.85)
BMI (kg/m^2^)	23.56 ± 3.52
BMI ranges (*n*, %) (*n* = 5,447)	
<18.5	418 (7.67)
18.5–23.9	2,670 (49.02)
24–27.9	1,835 (33.69)
≥28	524 (9.62)
Death (*n*, %) (*n =* 5215)	180 (3.45)
Length of hospital stay (days)	9.79 ± 7.56
Number of non-scheduled rehospitalizations 90 days after discharge (*n*, %) (*n =* 5139)	742 (14.44)
Initial biochemical data	
Hemoglobin (g/l)	122.68 ± 23.86
Serum albumin (g/l)	36.48 ± 7.48
Blood urea nitrogen (BUN, mmol/l)	7.37 ± 18.19
Creatinine (μmol/l)	87.38 ± 94.60
Department (*n*, 100%)	
Surgical	1,978 (35.86)
Medicine	2,211 (40.08)
Neurology	511 (9.26)
Orthopedics	500 (9.06)
ICU	316 (5.73)
Province or municipality/city (*n*, 100%)	
Sichuan province	1,584 (28.72)
Heilongjiang province	287 (5.20)
Hubei province	836 (15.16)
Beijing municipality/city	742 (13.45)
Qinghai province	758 (13.74)
Zhejiang province	1,309 (23.73)

*Data presented as mean ± standard deviation or frequency (%), as appropriate*.

*Some data were missing in the calculations*.

*MNA-SF, Mini Nutritional Assessment Short-Form; BMI, body mass index*.

**Table 2:** Data for hospitalizations, analyzed according to MNA-SF score.

**Parameter**	**Malnourished (*N =* 656)**	**Malnutrition risk (*N =* 1892)**	**Normal (*N =* 2968)**	***P*-Value**
Age (years)	73.34 ± 6.02	73.16 ± 6.01	72.18 ± 5.66	<0.001
Age ranges				<0.001
<75	413 (62.96)	1,248 (65.96)	2,138 (72.04)	
≥75	243 (37.04)	644 (34.04)	830 (27.96)	
Sex (*n*, %)				<0.001
Male gender	358 (54.57)	1,101 (58.19)	1,838 (61.93)	
Female gender	298 (45.43)	791 (41.81)	1,130 (38.07)	
Education (*n*, %) (*n =* 5,514)				<0.001
University	76 (11.62)	219 (11.58)	459 (15.46)	
Middle school	223 (34.10)	708 (37.42)	1,272 (42.86)	
Primary school	186 (28.44)	615 (32.51)	843 (28.40)	
Illiterate	169 (25.84)	350 (18.50)	394 (13.27)	
Marital status (*n*, %) (*n =* 5510)				<0.001
Married	568 (86.98)	1,652 (87.41)	2,744 (92.48)	
Divorced or widowed	85 (13.02)	238 (12.59)	223 (7.52)	
Ethnicity (*n*, %)				<0.001
Han	598 (91.16)	1,776 (93.87)	2,847 (95.92)	
Others	58 (8.84)	116 (6.13)	121 (4.08)	
Smoking (*n*, %)				<0.05
Current smoker	53 (8.08)	239 (12.63)	349 (11.76)	
Former smoker	144 (21.95)	425 (22.46)	717 (24.16)	
Non-smoker	459 (69.97)	1,228 (64.90)	1,902 (64.08)	
Alcohol consumption (*n*, %)				0.06
Current drinker	51 (7.77)	209 (11.05)	426 (14.35)	
Former drinker	85 (12.96)	265 (14.01)	351 (11.83)	
Non-drinker	520 (79.27)	1,418 (74.95)	2,191 (73.82)	
BMI (kg/m^2^)	20.47 ± 3.28	22.05 ± 3.25	24.97 ± 2.94	<0.001
BMI ranges (*n*, %) (*n =* 5447)				0.001
<18.5	187 (29.92)	231 (12.42)	0	
18.5–23.9	345 (55.20)	1,179 (63.39)	1,146 (38.69)	
24–27.9	78 (12.48)	361 (19.41)	1,396 (47.13)	
≥28	15 (2.40)	89 (4.78)	420 (14.18)	
Death (*n*, %) (*n =* 5215)	61 (10.29)	84 (4.72)	35 (1.23)	<0.001
Length of hospital stay (days)	12.58 ± 9.25	11.52 ± 8.54	9.87 ± 7.49	<0.001
Number of non-scheduled rehospitalizations 90 days after discharge (*n*, %) (*n =* 5139)	106 (18.76)	295 (16.92)	341 (12.05)	<0.001
Initial biochemical data				
Hemoglobin (g/l)	112.36 ± 25.89	120.27 ± 24.44	126.51 ± 22.08	<0.001
Serum albumin (g/l)	34.37 ± 7.26	35.96 ± 7.07	37.29 ± 7.65	<0.001
Blood urea nitrogen (BUN, mmol/l)	8.40 ± 18.76	7.54 ± 21.20	7.04 ± 15.83	0.41
Creatinine (μmol/l)	91.74 ± 100.79	91.66 ± 105.22	83.69 ± 85.46	<0.001

*Data presented as mean ± standard deviation or frequency (%), as appropriate*.

*Some data were missing in the calculations*.

*MNA-SF, Mini Nutritional Assessment Short-Form; BMI, body mass index*.

### Mortality

Table 1 displays mortality among the study participants. Overall, death occurred in 3.45% of study participants. In Table 2, the number of patients who died who were malnourished, at risk of malnutrition, and normal was 61 (10.29%), 84 (4.72%), and 35 (1.23%), respectively (*P* < 0.001). MNA-SF scores 0–7 (OR 5.738, 95% CI 3.473 to 9.48; *P* < 0.001) were associated with a six-fold higher likelihood of death and scores 8–11 (OR 3.283, 95% CI 2.126–5.069) with a three-fold higher likelihood of death, compared with MNA-SF scores 12–14 in the logistic regression model, after adjusting for potential confounders (Table 3). However, hemoglobin (OR 0.992, 95% CI 0.985–0.998; *P* < 0.05) and serum albumin (OR 0.927, 95% CI 0.902–0.951; *P* < 0.001) had a lower likelihood of increased mortality. Additionally, female patients had a lower likelihood of death than male patients (OR 0.646, 95% CI 0.433–0.962; *P* < 0.05). A higher level of education (OR 0.484, 95% CI 0.247–0.947; *P* < 0.05) was statistically associated with a decline in the risk of death, in comparison with illiterate patients.

**Table 3:** Prevalence of measured outcomes in relation to nutritional status and clinical parameters analyzed.

**Parameter which acted as a risk factor for measured outcome**	**Death^a^ Odds ratio, (95% CI)**	**Length of hospital stay^b^ Coefficients (95% CI)**	**90-day readmission^a^ dds ratio, (95% CI)**
Malnourished (0–7)	5.738 (3.473, 9.48)^***^	0.2807 (0.0294, 0.5320)^*^	1.393 (1.052, 1.843)
Malnutrition risk (8–11)	3.283 (2.126, 5.069)	0.2574 (0.0863, 0.4285)^**^	1.356 (1.124, 1.636)
Normal (12-14)	1.0 (Ref.)	0 (Ref.)	1.0 (Ref.)
BMI			
<18.5	1.448 (0.939, 2.233)	−0.2317 (−0.5197, 0.0562)	0.747 (0.543, 1.027)
18.5–23.9	1.0 (Ref.)	0 (Ref.)	1.0 (Ref.)
24–27.9	1.254 (0.837, 1.878)	−0.2737 (−0.4558, −0.0916)^**^	0.723 (0.594, 0.881)^*^
≥28	0.877 (0.392, 1.96)	−0.0185 (−0.2926, 0.2556)	0.974 (0.723, 1.313)
Hemoglobin (mg/dl)	0.992 (0.985, 0.998)^*^	−0.0081 (−0.0114, −0.0047)^***^	0.99 (0.986, 0.993)^***^
Serum albumin (g/l)	0.927 (0.902, 0.951)^***^	−0.0051 (−0.0164, 0.0061)	0.994 (0.982, 1.007)
Blood urea nitrogen (BUN, mg/dl)	1.004 (0.998, 1.01)	−0.0003 (−0.0021, 0.0015)	1.001 (0.997, 1.005)
Creatinine (μmol/l)	1.001 (1.000, 1.002)	0.0002 (−0.0005, 0.0009)	1.000 (0.999, 1.001)
Age (years)			
<75	1.0 (Ref.)	0 (Ref.)	1.0 (Ref.)
≥75	1.336 (0.962, 1.854)	−0.2592 (−0.4294, −0.0891)^**^	0.733 (0.609, 0.882)^**^
Sex			
Male gender	1.0 (Ref.)	0 (Ref.)	1.0 (Ref.)
Female gender	0.646 (0.433, 0.962)^*^	−0.1262 (−0.3243, 0.0718)	0.848 (0.683, 1.053)
Education			
University	0.484 (0.247, 0.947)^*^	−0.2416 (−0.5324, 0.0491)	0.746 (0.544, 1.024)
Middle school	0.846 (0.538, 1.328)	−0.0275 (−0.2463, 0.1914)	0.968 (0.76, 1.232)
Primary school	0.953 (0.606, 1.5)	−0.1392 (−0.3686, 0.0901)	0.841 (0.654, 1.082)
Illiterate	1.0 (Ref.)	0 (Ref.)	1.0 (Ref.)
Marital status			
Married	1.0 (Ref.)	0 (Ref.)	1.0 (Ref.)
Divorced or widowed	0.844 (0.496, 1.437)	−0.2380 (−0.5286, 0.0525)	0.765 (0.56, 1.044)
Ethnicity			
Han	1.0 (Ref.)	0 (Ref.)	1.0 (Ref.)
Others	1.041 (0.544, 1.991)	0.1661 (−0.1600, 0.4922)	1.246 (0.868, 1.788)
Smoking			
Current smoker	0.748 (0.411, 1.361)	0.0606 (−0.2119, 0.3331)	1.079 (0.802, 1.452)
Former smoker	0.993 (0.645, 1.529)	0.4041 (0.1994, 0.6088)^***^	1.632 (1.301, 2.047)^***^
Non-smoker	1.0 (Ref.)	0 (Ref.)	1.0 (Ref.)
Alcohol drinking			
Current drinker	0.54 (0.287, 1.018)	−0.5579 (−0.8452, −0.2706)^***^	0.52 (0.383, 0.707)^***^
Former drinker	0.89 (0.541, 1.463)	0.0534 (−0.1656, 0.2724)	1.072 (0.838, 1.371)
Non-drinker	1.0 (Ref.)	0 (Ref.)	1.0 (Ref.)

*** *P < 0.001*,

** *P < 0.01*,

* *P < 0.05*.

a *Logistic regression models*.

b *Negative binomial regression model*.

*BMI, body mass index; CI, confidence interval*.

### Length of Stay (LoS)

Table 2 also indicates the LoS among the patients, with an average LoS in the group with MNA-SF scores 0–7 of 12.58 ± 9.25 days, 11.52 ± 8.54 days in the group with MNA-SF scores 8–11, and 9.87 ± 7.49 in the group with MNA-SF scores 12–14. In Table 3, after adjusting for potential confounders in the negative binomial regression model, a low MNA-SF score of 0–7 (regression coefficient 0.2807, 95% CI 0.0294–0.5320; *P* < 0.05) and a score of 8–11 (0.2574, 95% CI 0.0863–0.4285; *P* < 0.01) was associated with a significantly (28.07 and 25.74%, respectively) higher likelihood of increased LoS compared with an MNA-SF score 12–14. Additionally, compared with nonsmokers, former smokers had a 40.41% (0.4041, 95% CI 0.1994–0.6088; *P* < 0.001) higher likelihood of increased LoS, whereas there was no significance among current smokers. However, BMI 24–27.9 kg/m^2^ (−0.2737, 95% CI −0.4558 to −0.0916; *P* < 0.01) and hemoglobin (−0.0081, 95% CI −0.0114 to −0.0047; *P* < 0.001) had a lower likelihood of increased LoS. Age 75 years and above (−0.2592, 95% CI −0.4294 to −0.0891; *P* < 0.01) and current alcohol consumption (−0.5579, 95% CI −0.8452 to −0.2706; *P* < 0.001) were also protective factors.

### Ninety-Day Readmission

Table 1 also shows that 742 participants (14.44%) had unscheduled rehospitalization 90 days after discharge. In Table 2, the number of patients with malnutrition, malnutrition risk, and normal nutritional status who had 90-day readmission was 106 (18.76%), 295 (16.92%), and 341 (12.05%), respectively (*P* < 0.001). In Table 3, MNA-SF scores 0–7 (OR 1.393, 95% CI 1.052–1.843) and 8–11 (OR 1.356, 95% CI 1.124–1.636) were associated with a nearly 1.5-fold higher likelihood of 90-day readmission, compared with MNA-SF scores 12–14 scores in the logistic regression model, after adjusting for potential confounders. Additionally, former smokers (OR 1.632, 95% CI 1.301–2.047; *P* < 0.001) had a higher risk of 90-day readmission than nonsmokers. However, BMI 24–27.9 kg/m^2^ (OR 0.723, 95% CI 0.594–0.881; *P* < 0.05), hemoglobin (OR 0.99, 95% CI 0.986–0.993; *P* < 0.001), age 75 years and above (OR 0.733, 95% CI 0.609–0.882; *P* < 0.01), and current drinking (OR 0.52, 95% CI 0.383–0.707; *P* < 0.001) were significantly associated with a lower risk of 90-day readmission in the multivariate model.

## Discussion

This study is among the first to examine the association between nutritional status and LoS, mortality, and readmission in a nationally representative sample of Chinese elder inpatients of tertiary hospitals. We found malnutrition (MNA-SF score 0–7) in 11.89% of all older hospitalized patients, and low MNA-SF scores were associated with increased average LoS, a greater likelihood of death, and 90-day readmission.

Studies are scarce in China that explore the associations between nutritional status and health outcomes among older inpatients. Adigüzel et al. (39) indicated that nutritional parameters and sociodemographic features could affect health-related quality of life and functional ability among home care patients in Turkey. A study conducted in Germany also suggested that malnutrition could increase the risk of morbidity, mortality, hospital LoS, and costs among surgical patients (22). Additionally, Valmorbida et al. (19) suggested that malnutrition is associated with an increased risk of hospital admission and death among the 144 Italian older adults. Therefore, these studies are in accordance with our results and emphasize the urgent need for physicians, nurses, and clinical institutions to be aware of the high prevalence of malnutrition in the elderly, periodic nutritional screening of geriatric inpatients is critical.

Malnutrition is a crucial condition among older inpatients, both as a cause and consequence of disease (14, 40). The prevalence of malnutrition is 46.19% in tertiary hospitals of China, which is higher than the results of a cross-sectional study conducted in nursing homes of Spain reporting that 40.1% of older residents had malnutrition or risk of malnutrition, as measured using the Mini Nutritional Assessment (MNA®) (41). Previous research has indicated that 38.2% of nursing home residents in Italy are malnourished or at risk of malnutrition according to the MNA-SF (19), and the prevalence of malnutrition and malnutrition risk among older patients with hip fracture in Israel ranges from 55.81% using the MNA-SF (10) to 56.59% (medium risk and nutritionally at risk, measured using the NRS-2002) in Hyogo, Japan (42). These differences are likely owing to a variety of factors, including differences in the nutritional assessment tools used, study participants, study design, study periods, and local factors, such as the health systems in different countries and the standards of medical treatment received (10, 15, 19, 21, 43).

In comparison with well-nourished patients, malnourished older patients in our study had higher mortality (OR 5.738, 95% CI 3.473–9.48; 10.29% vs. 1.23%), longer LoS (0.2807, 95% CI 0.0294–0.5320; *P* < 0.05; 12.58 ± 9.25 vs. 9.87 ± 7.49) and were more likely to be readmitted within 90 days (OR 1.393, 95% CI 1.052–1.843; 18.76% vs. 12.05%). In line with our results, Lim et al. (31) suggested that approximately 29% of malnourished Singaporean patients had a longer hospital stay (6.9 ± 7.3 days vs. 4.6 ± 5.6 days, *P* < 0.001) and were more likely to be readmitted within 15 days (adjusted relative risk = 1.9, 95% CI 1.1–3.2, *P*=0.025) than well-nourished patients (31). Agarwal et al. (44) suggested that the 90-day in-hospital mortality in malnourished patients was around 1.09–3.34 times that of well-nourished patients, readmission rates and mean LoS among the malnourished patients were 36% and 15 days, respectively. These differences might result from the demographic characteristics of study participants and different follow-up periods after discharge. These findings confirm that early and compulsory nutritional assessment to identify malnutrition or malnutrition risk in elder inpatients is critical and may help in targeting interventions to tackle malnutrition (16).

Despite the association between malnutrition assessed by the MNA-SF and adverse clinical outcomes, hemoglobin level was associated with the hospital LoS, mortality, and 90-day readmission, after adjusting for confounders in our sample. These results are in line with the current literature (45–47) that low hemoglobin is associated with adverse clinical outcomes among older adults. Moreover, previous research indicates that the Geriatric Nutrition Risk Index (GNRI) (42) combined with biochemical objective indicators, such as albumin, can be used to identify and diagnose malnutrition and can better predict 30-day mortality rate in older patients (48). In addition, Hong et al. (49) and Pérez-Ros et al. (50) indicated that compared with the robust, levels of hemoglobin showed lower values among the prefrail and frail group, and nutritional markers may be used for the evaluation of adverse clinical outcomes in older patients. Therefore, our study suggests that the MNA-SF may be used in combination with hemoglobin levels to predict clinical outcomes among older inpatients. Although further evaluations are needed to clarify this, we believe that such findings may have concrete, helpful implications in the clinical practice.

Serum albumin is a protective factor of death among this study population. Only 11.89% of older inpatients had low serum albumin [cut-off of 35 g/L (19)] in our sample. Besides, Bouillanne et al. suggested that serum albumin lower than 35 g/L was associated with a higher risk of death (51). Therefore, during nutritional assessment in older inpatients, we might take albumin values into consideration, which may inform prognosis and hospital services planning.

Concerning BMI <18.5 kg/m^2^ as an indicator of undernutrition, we did not find significant associations with LoS, mortality or readmission, which differs from reports that individuals with BMI <25 kg/m^2^ had a higher risk of death than those who were overweight or obese (19). Interestingly, among BMI categories, only BMI 24–27.9 kg/m^2^ was significantly associated with a lower LoS and risk of 90-day readmission than BMI 18.5–23.9 kg/m^2^. This can hypothetically indicate that older overweight inpatients have significantly higher muscle mass than their normal counterparts. Thus, characteristics related to overweight and higher muscle mass could introduce a positive association for LoS and readmission (52). However, other relevant clinical data were lacking, including muscle mass in older inpatients; therefore, prospective studies with more sophisticated assessments are needed to confirm our findings.

In addition to the nutritionally related parameters assessed, former smokers were associated with a 40.41% higher likelihood of increased LoS and 90-day readmission than nonsmokers, which further supports previous research findings (14, 53, 54). However, current alcohol consumption and age ≥75 years were protective factors for LoS and readmission. This can possibly be explained by evidence that older people who consume small to moderate amounts of alcohol [1–25 g/day (55)] are more likely to maintain functional status and mobility than nondrinkers (32, 56); limited alcohol consumption has been associated with a decreased risk of adverse clinical outcomes (57). Another possible explanation is that compared with inpatients aged 75 years and above, those younger than 75 years may be able to withstand surgical stresses, and underwent more surgical treatment, which increases the risk of medical complications and longer LoS (58, 59).

Older female inpatients had a lower likelihood of death than male inpatients, and higher education level was significantly associated with a lower mortality risk than low education levels. The clinical phenomenon that female individuals survive longer than male adults is well described (57, 60), and well-educated older people may better comprehend the importance of nutrient intake, daily exercise, and disease prevention (61, 62).

The main limitations of our study include the limited follow-up; investigations with a longer duration are needed to better clarify the present findings. A further limitation of this paper is not having assessed all nutritional parameters over time; we only used the MNA-SF with a few anthropometric measures and limited biochemical data. Moreover, the older inpatients enrolled in our study were all from tertiary hospitals and only one hospital in each province or municipality/city, patients may have more complex clinical features, which may limit the generalizability of our results to different settings. Additionally, patients are taken from multiple hospitals or wards with differing baselines, the severity of the comorbidity along with different characteristics of the patients may influence the mortality rate and length of stay. The patients enrolled in this study were relatively young, 68.87% of the patients were <75 years old, which also limited the generalizability of this study. ICU patients have a higher mortality rate compared to general wards, it is meaningful to explore the nutritional status and adverse clinical outcomes among the ICU older inpatients. We are going to conduct this study in the future. Besides, our use of a limited number of nutritional screening tools restricted the comparison of our results with those of other studies, and the nutritional status was analyzed using the original MNA-SF which was derived based on Western populations, whereas the recent studies conducted in Taiwan of China have resulted in a modified MNA-SF, we will use this modified MNA-SF for further study (63, 64). Finally, the study participants were hospitalized in multiple wards or departments; we did not analyze the medical and nursing care received by patients, nor did we analyze the reason for the hospital admission. The patients enrolled in the present study were older adults from surgical, neurology, orthopedics departments, internal medicine, and ICU of selected hospitals, thus there would be great differences in nutritional status among these participants who have these health conditions, have chronic diseases, or undergoing surgery could be associated with poor nutritional status among hospitalized older people. To be meaningful, we will learn from previous research and results will be analyzed according to the like-kind of patients only (65, 66). Chronic diseases including cancer, diabetes, and cardiovascular diseases could be associated with poor nutritional status among elder patients, whereas we did not analyze the impact of chronic diseases in this study. There is a lack of other relevant clinical data, including albumin data, comorbidities, clinical severity of disease, comprehensive geriatric assessment, sarcopenia, the fat fold, upper arm circumference, and thigh circumference, because these features were not recorded in the first place, which is an inherent drawback of retrospective analysis of a prospectively collected database. Prospective studies with more sophisticated assessments are needed in the future.

## Conclusions

The findings of this study suggested that malnutrition increases the risk of hospital LoS, mortality, and 90-day readmission among inpatients, in comparison with well-nourished inpatients. Use of nutritional assessment tools in all hospitalized Chinese patients is needed. The MNA-SF in combination with hemoglobin level may be used to identify a high risk of adverse clinical outcomes among older inpatients and may be a good predictor of patient outcomes. These findings could have major importance for the planning of hospital services, discharge planning, and post-discharge care.

## Data Availability Statement

The raw data supporting the conclusions of this article will be made available by the authors, without undue reservation.

## Ethics Statement

The studies involving human participants were reviewed and approved by Peking Union Medical College Hospital (S-K540). The patients/participants provided their written informed consent to participate in this study.

## Author Contributions

XWu: study concept and design. HL, JJia, and TX: analysis and interpretation of data. HL: editing of the manuscript and drafting of tables. XWu and HL: critical review of the manuscript for important intellectual content. MZ, XWe, JJin, HW, DL, XS, and SZ: patient recruitment, data collection, and manuscript editing. All authors critically reviewed and approved the manuscript before it was submitted.

## Funding

The Special Research Fund for Central Universities, Peking Union Medical College [Grant Number 2018PT33001] supported this study but had no role in study design or data collection, analysis, and interpretation, or manuscript conception and writing.

## Conflict of Interest

The authors declare that the research was conducted in the absence of any commercial or financial relationships that could be construed as a potential conflict of interest.

## Publisher's Note

All claims expressed in this article are solely those of the authors and do not necessarily represent those of their affiliated organizations, or those of the publisher, the editors and the reviewers. Any product that may be evaluated in this article, or claim that may be made by its manufacturer, is not guaranteed or endorsed by the publisher.

## Abbreviations

MNA-SF: Mini Nutritional Assessment Short-Form
BMI: body mass index
SD: standard deviation
CI: confidence interval
ICU: intensive care unit
CRF: Case Report Form.
